# Effect of chronic kidney disease on outcomes of total joint arthroplasty: a meta-analysis

**DOI:** 10.1186/s43019-020-0029-8

**Published:** 2020-02-12

**Authors:** Chang-Wan Kim, Hyun-Jung Kim, Chang-Rack Lee, Lih Wang, Seung Joon Rhee

**Affiliations:** 1 Department of Orthopedic Surgery, Inje University Busan Paik Hospital, 75, Bokji-ro, Busanjin-gu, Busan, 47392 Republic of Korea; 2 Department of Preventive Medicine, Korea University College of Medicine, Seoul, Republic of Korea; 3 Department of Orthopedic Surgery, Dong-A University College of Medicine, Busan, Republic of Korea; 4 Department of Orthopedic Surgery, Biomedical Research Institute, Pusan National University Hospital, Busan, Republic of Korea

**Keywords:** Chronic kidney disease, Dialysis, Arthroplasty, Outcomes, Morbidity, Mortality

## Abstract

**Background:**

This meta-analysis was conducted to evaluate the differences in preoperative comorbidities, postoperative mortality, the rate of periprosthetic joint infection (PJI), and revision rate after total joint arthroplasty (TJA) between patients with chronic kidney disease (CKD)(CKD group) and patients with normal kidney function (non-CKD group).

**Methods:**

We searched MEDLINE, EMBASE, and the Cochrane Library for studies assessing the effect of CKD on TJA outcome. This meta-analysis included studies that (1) compared the outcomes of TJA between the CKD and non-CKD groups; (2) compared the outcomes of TJA based on CKD stage; and (3) evaluated the risk factors for morbidity or mortality after TJA. We compared the mortality, PJI, and revision rate between CKD and non-CKD groups, and between dialysis-dependent patients (dialysis group) and non-dialysis-dependent patients (non-dialysis group).

**Results:**

Eighteen studies were included in this meta-analysis. In most studies that assessed preoperative comorbidities, the number and severity of preoperative comorbidities were reported to be higher in the CKD group than in the non-CKD group. The risk of mortality was found to be higher in the CKD and dialysis groups compared with the respective control groups. In the studies based on administrative data, the unadjusted odds ratio (OR) of PJI was significantly higher in the CKD group than in the non-CKD group; however, no significant difference between the groups was noted in the adjusted OR. After total hip arthroplasty (THA), the risk of PJI was higher in the dialysis group than in the non-dialysis group. No significant difference was noted between the groups in the rate of PJI following total knee arthroplasty. The revision rate did not significantly differ between the CKD and non-CKD groups in the studies that were based on administrative data. However, the unadjusted OR was significantly higher in the dialysis group than in the non-dialysis group.

**Conclusions:**

Preoperative comorbidities and mortality risk were higher in the CKD and dialysis groups than in their respective control groups. The risk of revision was greater in the dialysis group than in the non-dialysis group, and the risk of PJI in the dialysis group became even greater after THA. Surgeons should perform careful preoperative risk stratification and optimization for patients with CKD scheduled to undergo TJA.

## Background

Total knee arthroplasty (TKA) or total hip arthroplasty (THA) is widely performed in patients with end-stage arthritis worldwide. Several authors have reported good long-term clinical outcomes and survivorship after total joint arthroplasty (TJA) in the lower extremities [1–3]. However, some patients experience surgery-related complications, such as surgical site infection and implant loosening after TJA, as well as multiple medical complications that can lead to serious results, such as death. Several studies have reported that poor clinical outcomes after TJA are related to various risk factors, including surgeon-related and implant-related factors [4–7]. Because TJA is mainly performed in elderly patients, it is important to consider patient-related factors, such as preoperative comorbidities, when determining postoperative clinical outcomes. Correlation has been reported between comorbidities, such as cardiovascular disease, kidney disease, liver disease, and diabetes mellitus (DM), and various complications including mortality and periprosthetic joint infection (PJI) [8–10]. Chronic kidney disease (CKD) is defined as a reduction in glomerular filtration rate, albumin excretion, or both. The reported global prevalence rate of CKD is 8–16%, and it is one of several comorbidities that may be present in patients undergoing TJA [11–14]. Renal osteodystrophy and long-term dialysis in CKD are associated with increased risk of joint arthropathy and osteonecrosis, which can increase the requirement for TJA [15–18]. As CKD is associated with long-term DM and hypertension, patients with these conditions are highly likely to have other comorbidities. It has been reported that DM, which is regarded as an important risk factor for CKD, is correlated with aseptic loosening and PJI [10, 19]. CKD is also known to be correlated with cardiovascular mortality [13]. Therefore, the management of patients with CKD after TJA must include careful observation and treatment.

Mathew et al. [20] reported that CKD is an independent risk factor for mortality in patients undergoing non-cardiac surgery. However, the effect of CKD on postoperative mortality and morbidity in patients who underwent TJA was not adequately investigated. Several studies reported correlation between CKD and TJA outcomes. Reports from earlier studies vary with regard to the effect of CKD on the risk of complications, such as mortality, PJI, and arthroplasty revision rate [15, 21–26]. However, no systematic review or meta-analysis of this relationship was conducted. Therefore, it is essential to perform a systematic review of the available studies on the effect of CKD on the clinical outcomes of TJA.

This meta-analysis was conducted to evaluate differences in preoperative comorbidities and postoperative mortality, rate of PJI, and revision rate after TJA between patients who have CKD (CKD group) and patients with normal kidney function group (non-CKD group). We hypothesized that the CKD group would have more preoperative comorbidities and greater risks of mortality, PJI, and revision after TJA than the non-CKD group.

## Methods

### Literature search and information sources

This study was implemented in accordance with the guidelines of the Preferred Reporting Items for Systematic Reviews and Meta-analyses statement and was based on the Cochrane review method. An independent medical librarian searched three databases (i.e., MEDLINE, EMBASE, and the Cochrane Library) from the dates of inception to 10 May 2019, to identify studies that evaluated the effect of CKD on the clinical outcomes of TJA. We used the following Medical Subject Heading (MeSH) terms and/or text words: (“Renal Insufficiency, Chronic”[Mesh] OR “Kidney Failure, Chronic”[Mesh] OR “Chronic Renal Insufficiencies”[TW] OR “Chronic Kidney Failure”[TW]) AND (“Arthroplasty, Replacement”[Mesh] OR “total joint arthroplasty”[TW] OR “Arthroplasty, Replacement, Knee”[Mesh] OR “Knee Replacement Arthroplasty”[TW] OR “Arthroplasty, Replacement, Hip”[Mesh] OR “Hip Replacement Arthroplasty”[TW]). The full search procedure is shown in the Additional file 1. After the initial database search, the references of relevant articles were manually searched to identify additional studies. There was no restriction on the language and year of publication. Because this study was a meta-analysis of published literature, permission from the institutional review board or informed consent was not required.

### Study selection

This meta-analysis included studies that met the following criteria: (1) they compared TJA outcomes between patients with CKD and patients with normal kidney function, (2) they compared TJA outcomes based on the CKD stage or implementation of dialysis, and (3) they evaluated risk factors for morbidity or mortality after TJA using CKD as a variable. Review articles, case reports, and studies on hemiarthroplasty, partial replacement arthroplasty, or revision TJA, and studies that did not compare the clinical outcomes of TJA between patients with CKD and patients with normal kidney function were excluded from this analysis. For studies containing insufficient data for the evaluation of TJA outcomes, we sent an e-mail to the authors; studies were excluded from this analysis if we did not receive a response or obtain the necessary data. Furthermore, only studies that used clear terminology on disease severity and chronicity, such as CKD, chronic renal disease or failure, dialysis, end-stage renal disease (ESRD), and moderate to severe renal disease, were included in this analysis. Those that did not clearly indicate the severity or chronicity, such as kidney disease or renal disease, and those without a description of the definition of kidney disease or renal disease in the text were excluded from the meta-analysis.

Two reviewers independently screened the titles and abstracts of the searched studies and selected relevant studies. The decision to include the studies screened by title in the meta-analysis was confirmed through full-text review.

### Assessment of methodological quality

Two reviewers independently evaluated the methodological quality of the selected studies using the Newcastle–Ottawa scale for nonrandomized studies in a systematic review and/or meta-analysis; it comprised the following three criteria: selection of the study groups (four numbered items), comparability of the groups (one numbered item), and ascertainment of either the exposure or outcomes of interest for case–control or cohort studies (three numbered items). The Newcastle–Ottawa scale awards stars to the items in each criterion, based on the level of bias; the maximum number of stars that can be acquired is nine.

### Data extraction

Using a predefined data extraction form, two reviewers independently extracted the following data from the included studies: first author, year of publication, study design, sample size, type of surgery (THA or TKA), average age at the time of surgery, average follow-up duration, preoperative comorbidity, mortality, infection, and revision. Data on PJI or deep infection were extracted. Data on wound problems, superficial infections, and surgical site infections were excluded.

### Statistical analysis

A meta-analysis was conducted on the postoperative outcomes (mortality, PJI, and revision) between the CKD and non-CKD groups. Although CKD is categorized into five stages (stages 1–5) based on the estimated glomerular filtration rate (eGFR), only stages 3, 4, and 5, which are characterized by eGFR less than 60 mL/min/1.73 m^2^, are considered as CKD. Dialysis is particularly vital for patients with stage 5 CKD (ESRD). To assess the impact of the severity of CKD on postoperative outcomes, a meta-analysis was also conducted between patients with CKD stage 5/ESRD and dialysis-dependent patients (dialysis group) and non-ESRD and non-dialysis-dependent patients (non-dialysis group). The dialysis and non-dialysis groups of patients with CKD were not compared among the patients with CKD, but they were compared among all the patients who received TJA. In other words, the non-dialysis group also included patients with CKD who did not undergo dialysis. If studies separately reported both early and late complications after TJA, but did not report the raw data, only the data on early complications were used in the meta-analysis.

The meta-analysis was conducted by distinguishing between studies based on administrative data and studies based on hospital data. In the present study, hospital data refers to clinical data (e.g., serum creatinine level) recorded over the course of the patient’s treatment in the hospital. In contrast, administrative data refers to registry data or claims data collected by government institutions and other organizations. A random effect model was used. The meta-analysis was conducted on the TJA outcomes reported by more than two studies. The odds ratio (OR) or hazard ratio (HR) of each outcome (mortality, PJI occurrence, and revision rate) and 95% confidence intervals (CIs) were used in the meta-analysis. For studies that did not report the outcome using the OR, the OR was computed on the basis of the raw data from the study. Heterogeneity was assessed using the *I*^*2*^ statistic: *I*^*2*^ of 25% was regarded as low heterogeneity, 50% was regarded as moderate heterogeneity, and 75% was regarded as high heterogeneity. Forest plots were used to represent the outcomes of each study, pooled estimates of effect, and overall summary effects: *p* values smaller than 0.05 were regarded as statistically significant. All statistical analysis was conducted using RevMan version 5.3 (Copenhagen, Nordic Cochrane Centre, The Cochrane Collaboration, 2014).

## Results

### Study selection

**Fig. 1:**
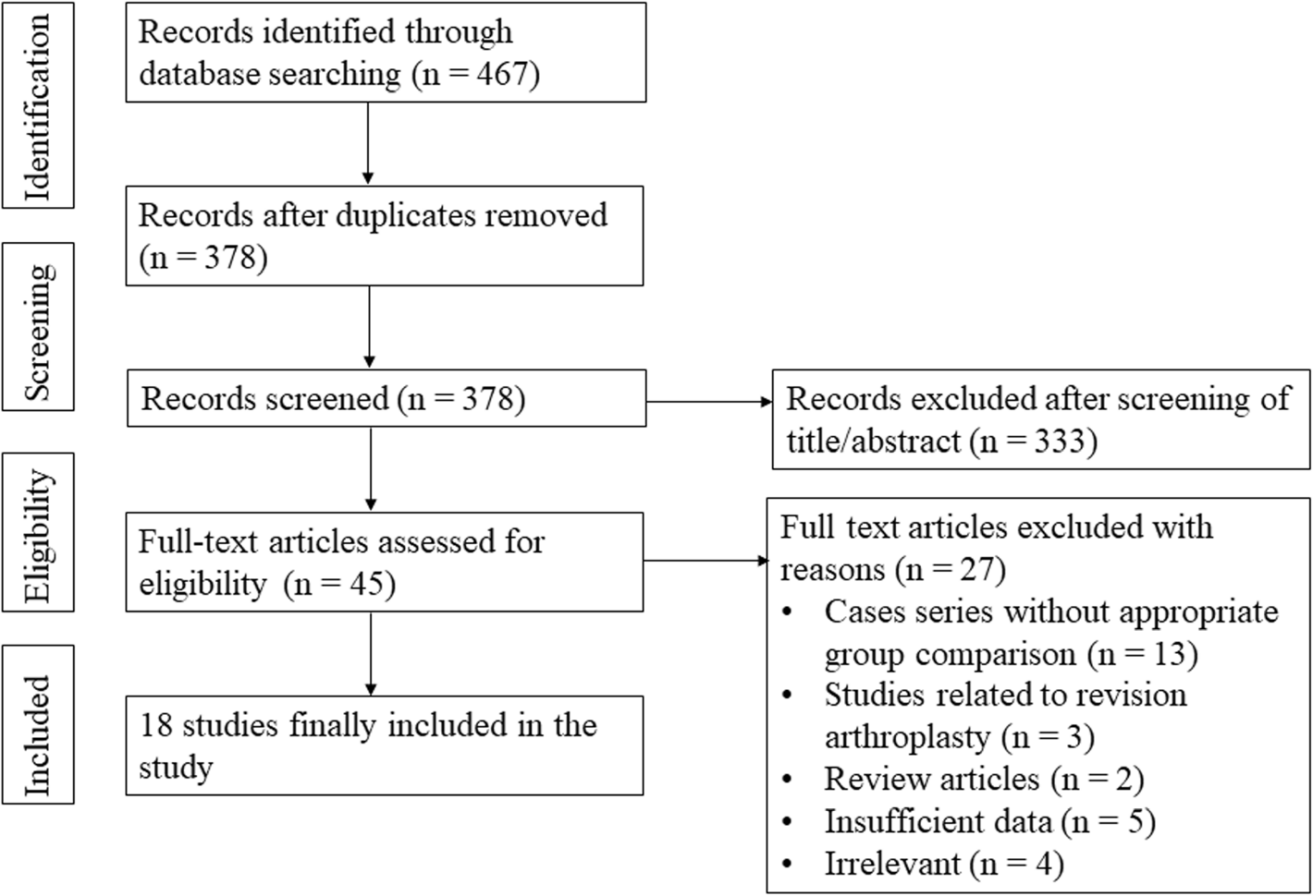
Preferred reporting items for systematic reviews and meta-analyses flow diagram

The study selection process is summarized in Fig. 1. Through literature searches, we found a total of 467 studies, including 80 in PubMed (MEDLINE), 371 in EMBASE, and 16 in the Cochrane Library. No further studies were found through manual searches. Following the removal of 89 duplicate studies, we conducted screening of the titles and abstracts of 378 studies and full-text review of the remaining 45 studies. Finally, 18 studies were included in this analysis.

### Study characteristics

Among the 18 studies included in this analysis, 12 studies [14, 22–32] were based on administrative data and 6 studies [15, 21, 33–36] on hospital data. Among the 18 studies, 4 studies [23, 27, 29, 31] reported the outcome of THA, 7 studies [22, 24, 28, 32–35] reported the outcome of TKA, and the remaining 7 studies [14, 15, 21, 25, 26, 30, 36] reported the outcomes of both THA and TKA.

The characteristics of the studies included are summarized in Table 1. In most of the studies that were based on hospital data, CKD was defined by the eGFR, which was defined on the basis of the Modification of Diet in Renal Disease equation [37], as follows: eGFR (mL/min/1.73 m^2^) = 186.3 × serum creatinine (mg/dL)^− 1.154^ × age (years)^− 0.203^ × (0.742 if the patient was female). CKD stage was categorized in accordance with the clinical guidelines of the National Kidney Foundation, as follows [37]: CKD stage 1 (normal) for eGFR ≥ 90 mL/min/1.73 m^2^; CKD stage 2 (mild) for eGFR 60–89 mL/min/1.73 m^2^; CKD stage 3 (moderate) for eGFR 30–59 mL/min/1.73 m^2^; CKD stage 4 (severe) for eGFR 15–29 mL/min/1.73 m^2^; and CKD stage 5 for eGFR < 15 mL/min/1.73 m^2^. Some studies indirectly reported the CKD stages of the research subjects using terminology such as chronic renal failure, ESRD, or moderate to severe renal disease. Other studies [38–40] did not clearly describe the definition or stage of CKD. One study defined CKD as preoperative creatinine > 1.5 mg/L [29]. In general, CKD is defined as eGFR < 60 mL/min/1.73 m2 [37]. In this study, patients with CKD stages 3, 4, and 5, and dialysis-dependent patients, were included in the CKD group, whereas patients with CKD stages 1 or 2 were included in the non-CKD group. For studies that were based on administrative data, the International Classification of Diseases, ninth revision, Clinical Modification [41] was used for the selection of research subjects. However, some studies did not contain specific descriptions of the method used for selection of research subjects.

**Table 1:** Study characteristics

Author	Year	Country	Study design	Database	Type of surgery	Mean age (years)	Total sample size	CKD stage	Number of cases in CKD group	Number of cases in control group	NOS
Bedard [27]	2018	USA	Retrospective case–control	Humana database	THA	N/A	17,695	N/A	2288	15,407	6
Bedard [28]	2018	USA	Retrospective case–control	Humana database	TKA	N/A	35,894	N/A	4551	31,343	6
Boniello [29]	2018	USA	Retrospective cohort study	ACS-NSQIP	THA	≥ 80		Preop Cr > 1.5 mg/L	1759	65,080	7
Cavanaugh [30]	2016	USA	Retrospective cohort study	NIS	THA, TKA	CKD: 71.9 non-CKD: 65.6	1,014,686	CKD stage 3–4, RT, dialysis, and ESRD	36,308	978,378	7
Deegan [21]	2014	USA	Retrospective cohort study	Geisinger Health System	THA, TKA	72	779	CKD stage 1, 2, 3	402	377	8
Erkocak [15]	2016	USA	Retrospective cohort study	Hospital data	THA, TKA	CKD: 67.8 Control: 67.2	1077	N/A	359	718	8
Kildow [31]	2017	USA	Retrospective cohort study	Medicare	THA	N/A	91,467	CKD stage 1–4, hemodialysis, RT	29,689	61,778	8
Kuo [33]	2017	Taiwan	Retrospective cohort study	Hospital data	TKA	CKD: 72.1 Non-CKD: 71.0	615	eGFR < 60	205	410	8
Kuo [22]	2017	Taiwan	Retrospective cohort study	NHIRD	TKA	CKD: 71.6 Non-CKD: 70.3	13,844	N/A	1459	12,385	8
Lizaur-Utrilla [34]	2016	Spain	Retrospective case–control	Hospital data	TKA	ESRD: 69.3 Control: 70.1	45	ESRD (dialysis or RT)	15	30	7
Marya [35]	2016	India	Retrospective case–control	Hospital data	Bilateral simultaneous TKA	65.8	556	Moderate-to-severe renal disease	11	N/A	7
McCleery [32]	2010	UK	Retrospective cohort study	Scottish Arthroplasty Project	TKA	N/A	59,288	Renal failure, RT, dialysis	3718	N/A	7
Miric [23]	2014	USA	Retrospective cohort study	TJRR	THA	66	18,663	CKD stage 3, 4, 5	1269	17,394	8
Miric [24]	2014	USA	Retrospective cohort study	TJRR	TKA	67	37,482	CKD stage 3, 4, 5	2686	34,796	8
Nikkinen [36]	2019	Finland	Retrospective cohort study	Hospital data	THA, TKA	Moderated to severe CKD: 81 Mild CKD: 77 Normal kidney function: 71	807	eGFR < 60	109	698	7
Patterson [25]	2018	USA	Retrospective cohort study	ACS-NSQIP	THA, TKA	N/A	THA:129370 TKA:214005	Dialysis	THA:306 TKA:339	THA:129064 TKA:213666	8
Ponnusamy [26]	2015	USA	Retrospective cohort study	NIS	THA, TKA	- THA Non-dialysis: 65.2 Dialysis: 63.2 - TKA Non-dialysis: 66.8 Dialysis: 66.7	THA: 2006522 TKA: 4182887	ESRD	THA:1251 TKA: 1683	THA: 2005271 TKA: 4181204	7
Warth [14]	2015	USA	Retrospective cohort study	ACS NSQIP	THA, TKA	Mild or normal CKD: 70.7 Moderate or severe CKD: 72.6	25,116	eGFR < 60	12,558	12,558	8

*ACS-NSQIP* American College of Surgeons-National Surgical Quality Improvement Program, *CKD* chronic kidney disease, *Cr* creatinine, *CRF* chronic renal failure, *eGFR* estimated glomerular filtration rate (mL/min/1.73 m^2^), *ESRD* end-stage renal disease, *N/A* not available, *NHIRD* National Health Insurance Research Database, *NIS* Nationwide inpatient sample, *NOS* Newcastle–Ottawa Scale (expressed as the number of stars assigned), *preop* preoperative, *RT* renal transplantation, *THA* total hip arthroplasty, *TJRR* Total Joint Replacement Registry, *TKA* total knee arthroplasty, *USA* United States of America, *UK* United Kingdom

### Preoperative morbidity

In 11 studies [14, 15, 22–26, 30, 33, 34, 36], the comorbidities or health status of patients in the CKD group before TJA were assessed using a variety of methods, including the American Society of Anesthesiologists (ASA) physical status score, the Charlson comorbidity index [42], and the Elixhauser comorbidity index [43]. Some studies reported only the frequencies of certain diseases, such as DM and cardiovascular disease. Hematologic status was evaluated on the basis of hemoglobin level, hematocrit level, white blood cell count, and platelet count.

Although the studies in this meta-analysis reported that different diseases had a relatively high prevalence in the CKD group compared with the prevalence in the non-CKD group, most reported that the CKD group had more preoperative comorbidities. The types of preoperative comorbidity evaluated differed among studies. In summary, cardiovascular disease, valvular disease, congestive heart failure, DM, rheumatoid arthritis, and peripheral vascular disease were more prevalent in the CKD group. Additionally, the CKD group had lower hemoglobin levels and higher ASA scores, and greater alcohol abuse and smoking frequency, relative to the non-CKD group.

### Mortality

**Fig. 2:**
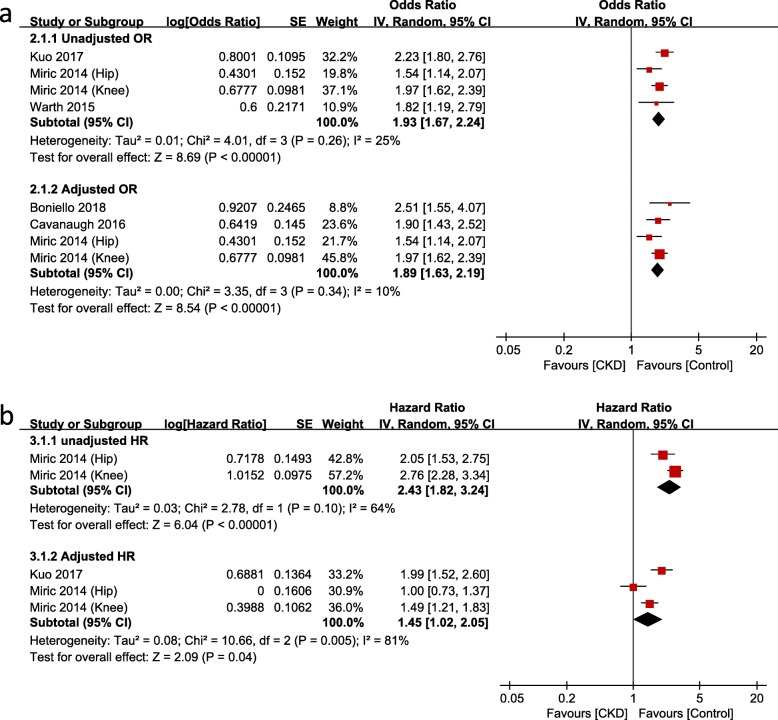
Meta-analysis of differences in mortality between chronic kidney disease (CKD) and non-CKD groups in studies based on administrative data. **a** odds ratio. **b** hazard ratio

**Fig. 3:**
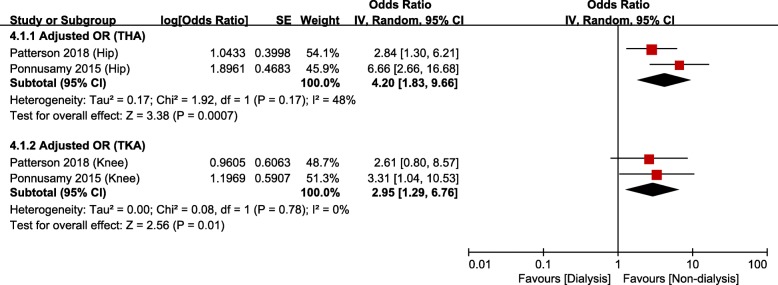
Meta-analysis of differences in mortality between dialysis and non-dialysis groups in studies based on administrative data

There were 13 studies [14, 15, 21–26, 29, 30, 34–36] that compared mortality after TJA between CKD and non-CKD groups or between dialysis and non-dialysis groups: among them, 8 studies were based on administrative data and 5 studies on hospital data. Meta-analysis of mortality in studies based on administrative data showed that the risk of mortality was significantly greater in the CKD group than that in the non-CKD group (Fig. 2a and b); the unadjusted OR was 1.93 (95% CI, 1.67–2.24; *p* < 0.00001; *I*^*2*^, 25%), and the adjusted OR was 1.89 (95% CI, 1.63–2.19; *p* < 0.00001; *I*^*2*^, 10%). The HR of mortality was also significantly higher in the CKD group than that in the non-CKD group. The unadjusted HR was 2.43 (95% CI, 1.82–3.24; *p* < 0.00001; *I*^*2*^, 64%), and the adjusted HR was 1.45 (95% CI, 1.02–2.05; *p* = 0.04; *I*^*2*^, 81%). The dialysis group had significantly greater mortality than the non-dialysis group after both THA (adjusted OR, 4.20; 95% CI, 1.83–9.66; *p* = 0.0007; *I*^*2*^, 48%) and TKA (adjusted OR, 2.95; 95% CI, 1.29–6.76; *p* < 0.01; *I*^*2*^, 0%; Fig. 3).

**Fig. 4:**
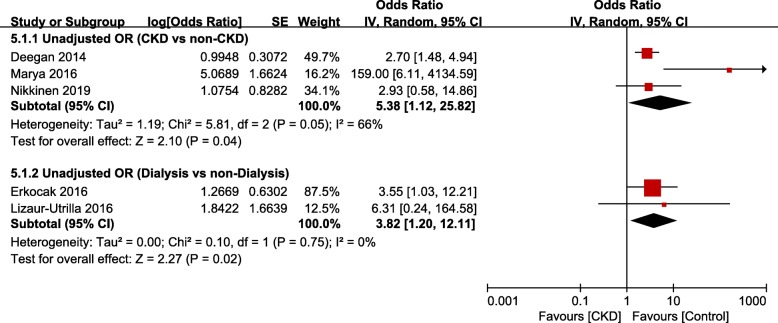
Meta-analysis of differences in mortality between chronic kidney disease (CKD) and non-CKD groups in studies based on hospital data

Meta-analysis of mortality in studies based on hospital data showed that respective risks of mortality were significantly greater in the CKD and dialysis groups than in the non-CKD (unadjusted OR, 5.38; 95% CI, 1.12–25.82; *p* = 0.004; *I*^*2*^, 66%) and non-dialysis groups (unadjusted OR, 3.82; 95% CI, 1.20–12.11; *p* = 0.02; *I*^*2*^, 0%; Fig. 4).

### Periprosthetic joint infection

**Fig. 5:**
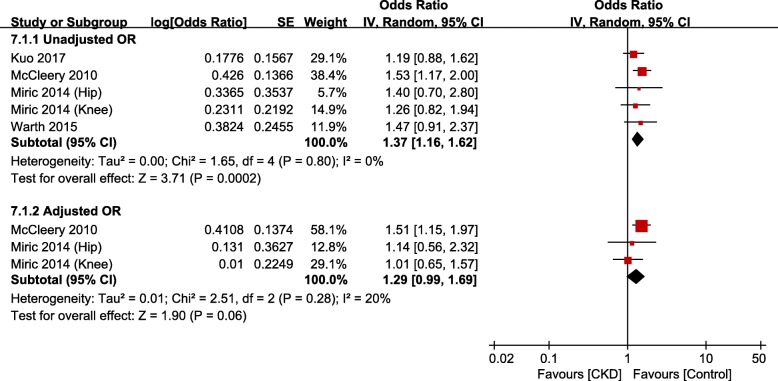
Meta-analysis of differences in periprosthetic joint infection (PJI) between chronic kidney disease (CKD) and non-CKD groups in studies based on administrative data

**Fig. 6:**
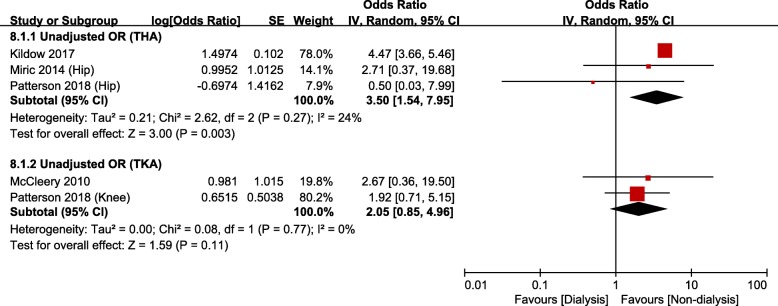
Meta-analysis of differences in periprosthetic joint infection (PJI) between dialysis and non-dialysis groups in studies based on administrative data

**Fig. 7:**
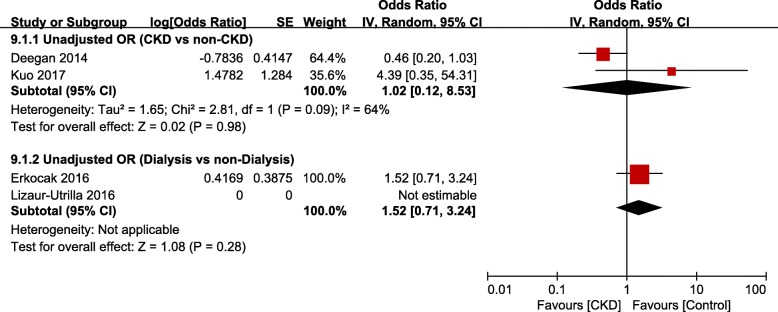
Meta-analysis of differences in periprosthetic joint infection (PJI) between chronic kidney disease (CKD) and non-CKD groups in studies based on hospital data

There were 11 studies [14, 15, 21–25, 31–34] that evaluated the occurrence of PJI after TJA in the CKD group: among them, 7 studies were based on administrative data and 4 studies on hospital data. Meta-analysis of studies based on administrative data showed that the risk of PJI was significantly greater in the CKD group than in the non-CKD group (unadjusted OR, 1.37; 95% CI, 1.16–1.62; *p* = 0.0002; *I*^*2*^, 0%; Fig. 5); however, the adjusted OR was not significant. After THA, the dialysis group had greater risk of PJI than the non-dialysis group (unadjusted OR, 3.50; 95% CI, 1.54–7.95; *p* = 0.003; *I*^*2*^, 24%; Fig. 6). The difference was not significant between groups after TKA. Meta-analysis of studies based on hospital data showed no significant differences between the CKD and non-CKD groups or between the dialysis and non-dialysis groups in terms of the occurrence of PJI (Fig. 7).

### Revision

**Fig. 8:**
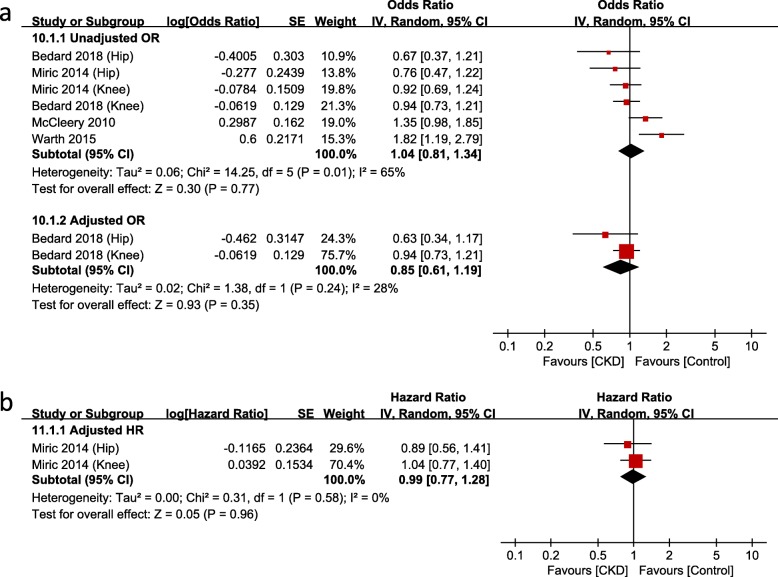
Meta-analysis of differences in revision surgery between chronic kidney disease (CKD) and non-CKD groups in studies based on administrative data

**Fig. 9:**
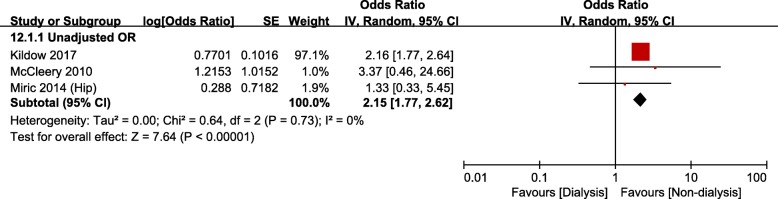
Meta-analysis of differences in revision surgery between dialysis and non-dialysis groups in studies based on administrative data

Nine studies [14, 21, 23, 24, 27, 28, 31, 32, 34] evaluated the revision rate after TJA in the CKD group. Among them, seven studies were based on administrative data and two studies on hospital data. However, meta-analysis could only be conducted on the studies based on administrative data: this analysis showed no significant differences in the revision rate between the CKD and non-CKD groups (Fig. 8a and b). The risk of revision in the ESRD/dialysis group was significantly greater than that in the non-dialysis group (unadjusted OR, 2.15; 95% CI, 1.77–2.62; *p* < 0.00001; *I*^*2*^, 0%; Fig. 9).

## Discussion

In this meta-analysis, patients with CKD had more preoperative comorbidities and more severe preoperative comorbidities and higher risk of mortality after TJA, compared with patients with normal kidney function. Patients with severe CKD (i.e., ESRD) and dialysis-dependent patients had more risk of PJI and revision surgery.

CKD is defined as kidney damage or glomerular filtration rate lower than 60 mL/min/1.73 m^2^ for ≥ 3 months; it poses a high risk of joint arthropathy or osteonecrosis secondary to renal osteodystrophy or long-term dialysis. Therefore, TJA is commonly performed in patients with CKD [11, 14, 15, 18, 44]. Because CKD is related to DM and hypertension, patients with CKD presumably have greater preoperative comorbidities and worse postoperative clinical outcomes, compared with patients with normal kidney function. A previous systematic review reported that CKD increases the risks of postoperative death or cardiovascular events among non-cardiac-surgery patients [20]. Although the effects of CKD on the outcomes of TJA have been reported in several studies, to the best of our knowledge, no systematic review or meta-analysis on this topic has yet been conducted. The value of the present meta-analysis is that it might enable surgeons to predict the prognosis of patients with CKD after TJA.

To assess the impact of CKD on TJA outcome, differences in the numbers and severities of comorbidities between CKD and non-CKD groups must be assessed. More preoperative comorbidities or more severe preoperative comorbidities will likely affect TJA outcome. Most studies in this meta-analysis reported that the CKD group had a relatively high prevalence of cardiovascular disease, DM, and peripheral vascular disease, which can be attributed to the relationships between CKD and DM and hypertension. Moreover, when compared with the non-CKD group, the CKD group exhibited higher prevalence of liver disease, rheumatoid arthritis, gout, alcohol abuse, and smoking, and had higher ASA and Charlson comorbidity scores and lower hemoglobin. Overall, although the tools used for evaluation of preoperative comorbidities varied among the studies, the CKD group had more comorbidities and more severe comorbidities than the non-CKD group.

Whether the presence of CKD is an independent risk factor for mortality in TJA is currently controversial. The current meta-analysis showed that the CKD and dialysis groups had greater mortality after TJA than the non-CKD and non-dialysis groups, respectively, regardless of the source of the data (hospital or administrative). More severe preoperative comorbidities are the greatest contributor to the greater mortality rate among patients with moderate to severe CKD, relative to patients with normal kidney function or mild CKD. After adjustment for diverse confounder effects, such as age and the presence of DM, however, it was difficult to conclude whether the presence of CKD itself was a risk factor for the high mortality rate. In this analysis, most of the studies based on administrative data controlled for confounders. However, most of the studies based on administrative data that we included in our meta-analysis controlled for confounders in their analyses. Furthermore, there was heterogeneity among the studies included. Considering these points, further studies are recommended that can clarify whether the presence of CKD is an independent predictor of mortality in TJA.

Many studies have reported correlation between the presence of CKD and the occurrence of infection after TJA. A number of factors, including nutritional deficiencies, anemia, metabolic imbalance, poor circulation, and reduced immunity, have been reported to affect the occurrence of PJI among patients with CKD [15, 30, 45]. Meta-analysis of studies based on administrative data showed that after THA, the CKD group had higher unadjusted odds of PJI than the non-CKD group, whereas the dialysis group had higher unadjusted odds of PJI than the non-dialysis group. Meta-analysis of studies based on hospital data showed no significant differences between the CKD and non-CKD groups. Considering the low incidence of PJI after TJA and the small number of studies based on hospital data, however, the results of meta-analysis of studies based on administrative data will have higher reliability. Therefore, the presence of CKD can be regarded as a risk factor for PJI after TJA.

In terms of revision, the dialysis group showed significantly higher odds of revision than the non-dialysis group in this study, whereas there was no significant difference between the CKD and non-CKD groups. The severity of CKD presumably affected the revision rate. Apart from medical comorbidities and the occurrence of PJI, the dialysis group showed a higher possibility of revision than the non-dialysis group for a variety of reasons, such as reduced osseointegration due to poor bone quality, implant loosening, or periprosthetic fracture [30, 46]. Considering the heterogeneity among studies included in this analysis and the short follow-up duration of the studies included, which interfered with evaluation of the revision rate, it is difficult to conclude whether the revision rate in patients with moderate CKD differs from that in patients with normal kidney function or mild CKD. To evaluate the effect of CKD on the rates of PJI and revision, high-quality studies that control for confounders are recommended in the future.

This study had several limitations. First, most studies were conducted in developed countries, such as the USA and European nations. Therefore, it is difficult to generalize the results of this study to developing countries. Second, the research subjects included in this study had various stages of CKD. Postoperative outcomes should be evaluated on the basis of CKD stage, to accurately assess the effect of the presence of CKD on TJA outcomes. However, most studies included in this meta-analysis did not present outcomes based on CKD stage. Nevertheless, depending on the severity of disease, TJA outcomes could be indirectly evaluated through the comparison of outcomes between dialysis and non-dialysis groups. Third, surgical types and techniques varied among studies. Although subgroup analysis was conducted on the basis of the type of surgery, it was insufficient to draw conclusions on correlation between the type of surgery and the outcomes reported. Last, on comparing the dialysis and non-dialysis groups, patients with CKD who did not receive dialysis were included in the non-dialysis group. This group distinction should be considered when interpreting the present results. Comparison within the CKD group between patients who underwent dialysis and those who did not could not be performed in the present study because of the lack of data.

## Conclusions

Preoperative comorbidity and mortality risk were greater in the CKD and dialysis groups than in their respective control groups. The risk of revision was greater in the dialysis group than in the non-dialysis group, and the risk of PJI in the dialysis group became even greater after THA. Surgeons should perform careful preoperative risk stratification and optimization for patients with CKD scheduled to undergo TJA.

## Supplementary information

**Additional file 1.** Search terms.

## Data Availability

Not applicable.

## Abbreviations

ASA: American Society of Anesthesiologists
CI: Confidence interval
CKD: Chronic kidney disease
DM: Diabetes mellitus
eGFR: Estimated glomerular filtration rate
ESRD: End-stage renal disease
HR: Hazard ratio
MeSH: Medical subject heading
OR: Odds ratio
PJI: Periprosthetic joint infection
THA: Total hip arthroplasty
TJA: Total joint arthroplasty
TKA: Total knee arthroplasty
