# Supplementary material for: Effect of chronic kidney disease on outcomes of total joint arthroplasty: a meta-analysis
Source: Knee Surg Relat Res. 2020 Feb 12;32:12. doi: 10.1186/s43019-020-0029-8 (PMC7219208; doi:10.1186/s43019-020-0029-8)
Supplement: Supplementary file 1 — Additional file 1. Search terms. [file 43019_2020_29_MOESM1_ESM.docx]

| **Database** | **Search** | **Search term** | **No. of studies (2019.5.10)** |
| --- | --- | --- | --- |
| PubMed | #1 | "Renal Insufficiency, Chronic"[Mesh] | 107,422 |
|  | #2 | "Chronic Renal Insufficiencies"[TW] OR "Renal Insufficiencies, Chronic"[TW] OR "Chronic Renal Insufficiency"[TW] OR "Kidney Insufficiency, Chronic"[TW] OR "Chronic Kidney Insufficiency"[TW] OR "Chronic Kidney Insufficiencies"[TW] OR "Kidney Insufficiencies, Chronic"[TW] OR "Chronic Kidney Diseases"[TW] OR "Chronic Kidney Disease"[TW] OR "Disease, Chronic Kidney"[TW] OR "Diseases, Chronic Kidney"[TW] OR "Kidney Disease, Chronic"[TW] OR "Kidney Diseases, Chronic"[TW] OR "Chronic Renal Diseases"[TW] OR "Chronic Renal Disease"[TW] OR "Disease, Chronic Renal"[TW] OR "Diseases, Chronic Renal"[TW] OR "Renal Disease, Chronic"[TW] OR "Renal Diseases, Chronic"[TW] | 58,271 |
|  | #3 | "Kidney Failure, Chronic"[Mesh] | 90,033 |
|  | #4 | "Kidney Failure, Chronic"[TW] OR "End-Stage Kidney Disease"[TW] OR "Disease, End-Stage Kidney"[TW] OR "End Stage Kidney Disease"[TW] OR "Kidney Disease, End-Stage"[TW] OR "Chronic Kidney Failure"[TW] OR "End-Stage Renal Disease"[TW] OR "Disease, End-Stage Renal"[TW] OR "End Stage Renal Disease"[TW] OR "Renal Disease, End-Stage"[TW] OR "Renal Disease, End Stage"[TW] OR "Renal Failure, End-Stage"[TW] OR "End-Stage Renal Failure"[TW] OR "Renal Failure, End Stage"[TW] OR "Renal Failure, Chronic"[TW] OR "Chronic Renal Failure"[TW] OR "ESRD"[TW] | 116,637 |
|  | #5 Combine | ((("Renal Insufficiency, Chronic"[Mesh]) OR (("Chronic Renal Insufficiencies"[TW] OR "Renal Insufficiencies, Chronic"[TW] OR "Chronic Renal Insufficiency"[TW] OR "Kidney Insufficiency, Chronic"[TW] OR "Chronic Kidney Insufficiency"[TW] OR "Chronic Kidney Insufficiencies"[TW] OR "Kidney Insufficiencies, Chronic"[TW] OR "Chronic Kidney Diseases"[TW] OR "Chronic Kidney Disease"[TW] OR "Disease, Chronic Kidney"[TW] OR "Diseases, Chronic Kidney"[TW] OR "Kidney Disease, Chronic"[TW] OR "Kidney Diseases, Chronic"[TW] OR "Chronic Renal Diseases"[TW] OR "Chronic Renal Disease"[TW] OR "Disease, Chronic Renal"[TW] OR "Diseases, Chronic Renal"[TW] OR "Renal Disease, Chronic"[TW] OR "Renal Diseases, Chronic"[TW]))) OR "Kidney Failure, Chronic"[Mesh]) OR (("Kidney Failure, Chronic"[TW] OR "End-Stage Kidney Disease"[TW] OR "Disease, End-Stage Kidney"[TW] OR "End Stage Kidney Disease"[TW] OR "Kidney Disease, End-Stage"[TW] OR "Chronic Kidney Failure"[TW] OR "End-Stage Renal Disease"[TW] OR "Disease, End-Stage Renal"[TW] OR "End Stage Renal Disease"[TW] OR "Renal Disease, End-Stage"[TW] OR "Renal Disease, End Stage"[TW] OR "Renal Failure, End-Stage"[TW] OR "End-Stage Renal Failure"[TW] OR "Renal Failure, End Stage"[TW] OR "Renal Failure, Chronic"[TW] OR "Chronic Renal Failure"[TW] OR "ESRD"[TW)) | 156,719 |
|  | #6 | "Arthroplasty, Replacement"[Mesh] | 50,002 |
|  | #7 | "Arthroplasty, Replacement"[TW] OR "Arthroplasties, Replacement"[TW] OR "Joint Prosthesis Implantation"[TW] OR "Implantation, Joint Prosthesis"[TW] OR "Implantations, Joint Prosthesis"[TW] OR "Joint Prosthesis Implantations"[TW] OR "Prosthesis Implantation, Joint"[TW] OR "Prosthesis Implantations, Joint"[TW] OR "Replacement Arthroplasty"[TW] OR "Joint Replacement"[TW] OR "Joint Replacements"[TW] OR "Replacement, Joint"[TW] OR "Replacements, Joint"[TW] OR "Replacement Arthroplasties"[TW] OR "Total Joint Replacement"[TW] OR "Joint Replacement, Total"[TW] OR "Joint Replacements, Total"[TW] OR "Replacement, Total Joint"[TW] OR "Replacements, Total Joint"[TW] OR "Total Joint Replacements"[TW] | 53,792 |
|  | #8 | "total joint arthroplasty"[TW] | 2,634 |
|  | #9 | "Arthroplasty, Replacement, Knee"[Mesh] | 21,390 |
|  | #10 | "Arthroplasty, Replacement, Knee"[TW] OR "Arthroplasties, Replacement, Knee"[TW] OR "Arthroplasty, Knee Replacement"[TW] OR "Knee Replacement Arthroplasties"[TW] OR "Knee Replacement Arthroplasty"[TW] OR "Replacement Arthroplasties, Knee"[TW] OR "Knee Arthroplasty, Total"[TW] OR "Arthroplasty, Total Knee"[TW] OR "Total Knee Arthroplasty"[TW] OR "Replacement, Total Knee"[TW] OR "Total Knee Replacement"[TW] OR "Knee Replacement, Total"[TW] OR "Knee Arthroplasty"[TW] OR "Arthroplasty, Knee"[TW] OR "Arthroplasties, Knee Replacement"[TW] OR "Replacement Arthroplasty, Knee"[TW] OR "Arthroplasty, Replacement, Partial Knee"[TW] OR "Unicompartmental Knee Arthroplasty"[TW] OR "Arthroplasty, Unicompartmental Knee"[TW] OR "Knee Arthroplasty, Unicompartmental"[TW] OR "Unicondylar Knee Arthroplasty"[TW] OR "Arthroplasty, Unicondylar Knee"[TW] OR "Knee Arthroplasty, Unicondylar"[TW] OR "Partial Knee Arthroplasty"[TW] OR "Arthroplasty, Partial Knee"[TW] OR "Knee Arthroplasty, Partial"[TW] OR "Unicondylar Knee Replacement"[TW] OR "Knee Replacement, Unicondylar"[TW] OR "Partial Knee Replacement"[TW] OR "Knee Replacement, Partial"[TW] OR "Unicompartmental Knee Replacement"[TW] OR "Knee Replacement, Unicompartmental"[TW] | 29,823 |
|  | #11 | "Arthroplasty, Replacement, Hip"[Mesh] | 24,740 |
|  | #12 | "Arthroplasty, Replacement, Hip"[TW] OR "Arthroplasties, Replacement, Hip"[TW] OR "Arthroplasty, Hip Replacement"[TW] OR "Hip Prosthesis Implantation"[TW] OR "Hip Prosthesis Implantations"[TW] OR "Implantation, Hip Prosthesis"[TW] OR "Implantations, Hip Prosthesis"[TW] OR "Prosthesis Implantation, Hip"[TW] OR "Prosthesis Implantations, Hip"[TW] OR "Hip Replacement Arthroplasty"[TW] OR "Replacement Arthroplasties, Hip"[TW] OR "Replacement Arthroplasty, Hip"[TW] OR "Arthroplasties, Hip Replacement"[TW] OR "Hip Replacement Arthroplasties"[TW] OR "Hip Replacement, Total"[TW] OR "Replacement, Total Hip"[TW] OR "Hip Replacements, Total"[TW] OR "Replacements, Total Hip"[TW] OR "Total Hip Replacements"[TW] OR "Total Hip Replacement"[TW] | 29,580 |
|  | #13 | "total hip arthroplasty"[TW] | 16,694 |
|  | #14 Combine | ((((((("Arthroplasty, Replacement"[Mesh]) OR (("Arthroplasty, Replacement"[TW] OR "Arthroplasties, Replacement"[TW] OR "Joint Prosthesis Implantation"[TW] OR "Implantation, Joint Prosthesis"[TW] OR "Implantations, Joint Prosthesis"[TW] OR "Joint Prosthesis Implantations"[TW] OR "Prosthesis Implantation, Joint"[TW] OR "Prosthesis Implantations, Joint"[TW] OR "Replacement Arthroplasty"[TW] OR "Joint Replacement"[TW] OR "Joint Replacements"[TW] OR "Replacement, Joint"[TW] OR "Replacements, Joint"[TW] OR "Replacement Arthroplasties"[TW] OR "Total Joint Replacement"[TW] OR "Joint Replacement, Total"[TW] OR "Joint Replacements, Total"[TW] OR "Replacement, Total Joint"[TW] OR "Replacements, Total Joint"[TW] OR "Total Joint Replacements"[TW]))) OR "total joint arthroplasty"[TW]) OR "Arthroplasty, Replacement, Knee"[Mesh]) OR (("Arthroplasty, Replacement, Knee"[TW] OR "Arthroplasties, Replacement, Knee"[TW] OR "Arthroplasty, Knee Replacement"[TW] OR "Knee Replacement Arthroplasties"[TW] OR "Knee Replacement Arthroplasty"[TW] OR "Replacement Arthroplasties, Knee"[TW] OR "Knee Arthroplasty, Total"[TW] OR "Arthroplasty, Total Knee"[TW] OR "Total Knee Arthroplasty"[TW] OR "Replacement, Total Knee"[TW] OR "Total Knee Replacement"[TW] OR "Knee Replacement, Total"[TW] OR "Knee Arthroplasty"[TW] OR "Arthroplasty, Knee"[TW] OR "Arthroplasties, Knee Replacement"[TW] OR "Replacement Arthroplasty, Knee"[TW] OR "Arthroplasty, Replacement, Partial Knee"[TW] OR "Unicompartmental Knee Arthroplasty"[TW] OR "Arthroplasty, Unicompartmental Knee"[TW] OR "Knee Arthroplasty, Unicompartmental"[TW] OR "Unicondylar Knee Arthroplasty"[TW] OR "Arthroplasty, Unicondylar Knee"[TW] OR "Knee Arthroplasty, Unicondylar"[TW] OR "Partial Knee Arthroplasty"[TW] OR "Arthroplasty, Partial Knee"[TW] OR "Knee Arthroplasty, Partial"[TW] OR "Unicondylar Knee Replacement"[TW] OR "Knee Replacement, Unicondylar"[TW] OR "Partial Knee Replacement"[TW] OR "Knee Replacement, Partial"[TW] OR "Unicompartmental Knee Replacement"[TW] OR "Knee Replacement, Unicompartmental"[TW]))) OR "Arthroplasty, Replacement, Hip"[Mesh]) OR (("Arthroplasty, Replacement, Hip"[TW] OR "Arthroplasties, Replacement, Hip"[TW] OR "Arthroplasty, Hip Replacement"[TW] OR "Hip Prosthesis Implantation"[TW] OR "Hip Prosthesis Implantations"[TW] OR "Implantation, Hip Prosthesis"[TW] OR "Implantations, Hip Prosthesis"[TW] OR "Prosthesis Implantation, Hip"[TW] OR "Prosthesis Implantations, Hip"[TW] OR "Hip Replacement Arthroplasty"[TW] OR "Replacement Arthroplasties, Hip"[TW] OR "Replacement Arthroplasty, Hip"[TW] OR "Arthroplasties, Hip Replacement"[TW] OR "Hip Replacement Arthroplasties"[TW] OR "Hip Replacement, Total"[TW] OR "Replacement, Total Hip"[TW] OR "Hip Replacements, Total"[TW] OR "Replacements, Total Hip"[TW] OR "Total Hip Replacements"[TW] OR "Total Hip Replacement"[TW]))) OR "total hip arthroplasty"[TW] | 72,292 |
|  | #15 Combine | #5 AND #14 | 143 |

| **Database** | **Search** | **Search term** | **No. of studies (2019.5.10)** |
| --- | --- | --- | --- |
| EMBASE | #1 | chronic kidney failure'/exp OR 'chronic kidney failure' | 136,508 |
|  | #2 | chronic renal insufficiencies' OR 'renal insufficiencies, chronic' OR 'chronic renal insufficiency'/exp OR 'chronic renal insufficiency' OR 'kidney insufficiency, chronic' OR 'chronic kidney insufficiency'/exp OR 'chronic kidney insufficiency' OR 'chronic kidney insufficiencies' OR 'kidney insufficiencies, chronic' OR 'chronic kidney diseases' OR 'chronic kidney disease'/exp OR 'chronic kidney disease' OR 'disease, chronic kidney' OR 'diseases, chronic kidney' OR 'kidney disease, chronic'/exp OR 'kidney disease, chronic' OR 'kidney diseases, chronic' OR 'chronic renal diseases' OR 'chronic renal disease'/exp OR 'chronic renal disease' OR 'disease, chronic renal' OR 'diseases, chronic renal' OR 'renal disease, chronic' OR 'renal diseases, chronic' | 153,757 |
|  | #3 | kidney failure, chronic'/exp OR 'kidney failure, chronic' OR 'end-stage kidney disease'/exp OR 'end-stage kidney disease' OR 'disease, end-stage kidney' OR 'end stage kidney disease'/exp OR 'end stage kidney disease' OR 'kidney disease, end-stage' OR 'end-stage renal disease'/exp OR 'end-stage renal disease' OR 'disease, end-stage renal' OR 'end stage renal disease'/exp OR 'end stage renal disease' OR 'renal disease, end-stage' OR 'renal disease, end stage' OR 'renal failure, end-stage' OR 'end-stage renal failure'/exp OR 'end-stage renal failure' OR 'renal failure, end stage' OR 'renal failure, chronic' OR 'chronic renal failure'/exp OR 'chronic renal failure' OR 'esrd'/exp OR 'esrd' | 196,228 |
|  | #4 Combine | chronic kidney failure'/exp OR 'chronic kidney failure' OR 'chronic renal insufficiencies' OR 'renal insufficiencies, chronic' OR 'chronic renal insufficiency'/exp OR 'chronic renal insufficiency' OR 'kidney insufficiency, chronic' OR 'chronic kidney insufficiency'/exp OR 'chronic kidney insufficiency' OR 'chronic kidney insufficiencies' OR 'kidney insufficiencies, chronic' OR 'chronic kidney diseases' OR 'chronic kidney disease'/exp OR 'chronic kidney disease' OR 'disease, chronic kidney' OR 'diseases, chronic kidney' OR 'kidney disease, chronic'/exp OR 'kidney disease, chronic' OR 'kidney diseases, chronic' OR 'chronic renal diseases' OR 'chronic renal disease'/exp OR 'chronic renal disease' OR 'disease, chronic renal' OR 'diseases, chronic renal' OR 'renal disease, chronic' OR 'renal diseases, chronic' OR 'kidney failure, chronic'/exp OR 'kidney failure, chronic' OR 'end-stage kidney disease'/exp OR 'end-stage kidney disease' OR 'disease, end-stage kidney' OR 'end stage kidney disease'/exp OR 'end stage kidney disease' OR 'kidney disease, end-stage' OR 'end-stage renal disease'/exp OR 'end-stage renal disease' OR 'disease, end-stage renal' OR 'end stage renal disease'/exp OR 'end stage renal disease' OR 'renal disease, end-stage' OR 'renal disease, end stage' OR 'renal failure, end-stage' OR 'end-stage renal failure'/exp OR 'end-stage renal failure' OR 'renal failure, end stage' OR 'renal failure, chronic' OR 'chronic renal failure'/exp OR 'chronic renal failure' OR 'esrd'/exp OR 'esrd' | 211,019 |
|  | #5 | replacement arthroplasty'/exp OR 'replacement arthroplasty' | 34,583 |
|  | #6 | arthroplasty, replacement'/exp OR 'arthroplasty, replacement' OR 'arthroplasties, replacement' OR 'joint prosthesis implantation' OR 'implantation, joint prosthesis' OR 'implantations, joint prosthesis' OR 'joint prosthesis implantations' OR 'prosthesis implantation, joint' OR 'prosthesis implantations, joint' OR 'replacement arthroplasty'/exp OR 'replacement arthroplasty' OR 'joint replacement'/exp OR 'joint replacement' OR 'joint replacements' OR 'replacement, joint'/exp OR 'replacement, joint' OR 'replacements, joint' OR 'replacement arthroplasties' OR 'total joint replacement'/exp OR 'total joint replacement' OR 'joint replacement, total' OR 'joint replacements, total' OR 'replacement, total joint' OR 'replacements, total joint' OR 'total joint replacements' | 94,842 |
|  | #7 | total joint arthroplasty'/exp OR 'total joint arthroplasty' | 3,071 |
|  | #8 | knee replacement'/exp OR 'knee replacement' | 31,997 |
|  | #9 | arthroplasty, replacement, knee'/exp OR 'arthroplasty, replacement, knee' OR 'arthroplasties, replacement, knee' OR 'arthroplasty, knee replacement' OR 'knee replacement arthroplasties' OR 'knee replacement arthroplasty'/exp OR 'knee replacement arthroplasty' OR 'replacement arthroplasties, knee' OR 'knee arthroplasty, total'/exp OR 'knee arthroplasty, total' OR 'arthroplasty, total knee' OR 'total knee arthroplasty'/exp OR 'total knee arthroplasty' OR 'replacement, total knee' OR 'total knee replacement'/exp OR 'total knee replacement' OR 'knee replacement, total'/exp OR 'knee replacement, total' OR 'knee arthroplasty'/exp OR 'knee arthroplasty' OR 'arthroplasty, knee'/exp OR 'arthroplasty, knee' OR 'arthroplasties, knee replacement' OR 'replacement arthroplasty, knee' OR 'arthroplasty, replacement, partial knee' OR 'unicompartmental knee arthroplasty'/exp OR 'unicompartmental knee arthroplasty' OR 'arthroplasty, unicompartmental knee' OR 'knee arthroplasty, unicompartmental' OR 'unicondylar knee arthroplasty'/exp OR 'unicondylar knee arthroplasty' OR 'arthroplasty, unicondylar knee' OR 'knee arthroplasty, unicondylar' OR 'partial knee arthroplasty' OR 'arthroplasty, partial knee' OR 'knee arthroplasty, partial' OR 'unicondylar knee replacement' OR 'knee replacement, unicondylar' OR 'partial knee replacement' OR 'knee replacement, partial' OR 'unicompartmental knee replacement'/exp OR 'unicompartmental knee replacement' OR 'knee replacement, unicompartmental' | 43,357 |
|  | #10 | hip replacement'/exp OR 'hip replacement' | 17,394 |
|  | #11 | total hip replacement'/exp OR 'hip replacement, total' OR 'replacement, total hip' OR 'hip replacements, total' OR 'replacements, total hip' OR 'total hip replacements' OR 'total hip replacement' OR 'hip total replacement' OR 'hip total replacements' OR 'hip total joint replacement' OR 'hip total joint replacements' OR 'replacement, hip total joint' OR 'replacements, hip total joint' OR ' hip replacement, total joint' OR ' hip replacements, total joint' OR 'hip total replacement arthroplasty' OR 'hip total replacement arthroplasties' OR 'hip total arthroplasty' OR 'hip total arthroplasties' OR 'arthroplasty, replacement, hip' OR 'arthroplasties, replacement, hip' OR 'arthroplasty, hip replacement' OR 'hip replacement, arthroplasty' OR 'hip replacement, arthroplasties' OR 'hip prosthesis implantation' OR 'hip prosthesis implantations' OR 'implantation, hip prosthesis' OR 'implantations, hip prosthesis' OR 'prosthesis implantation, hip' OR 'prosthesis implantations, hip' OR 'hip replacement arthroplasty' OR 'replacement arthroplasties, hip' OR 'replacement arthroplasty, hip' OR 'arthroplasties, hip replacement' OR 'arthroplasty, hip replacement' OR 'hip replacement arthroplasties' OR 'total hip arthroplasty' OR 'total hip arthroplasties' OR 'total replacement hip arthroplasty' OR 'total replacement hip arthroplasties' | 30,940 |
|  | #12 Combine | replacement arthroplasty'/exp OR 'replacement arthroplasty' OR 'arthroplasty, replacement'/exp OR 'arthroplasty, replacement' OR 'arthroplasties, replacement' OR 'joint prosthesis implantation' OR 'implantation, joint prosthesis' OR 'implantations, joint prosthesis' OR 'joint prosthesis implantations' OR 'prosthesis implantation, joint' OR 'prosthesis implantations, joint' OR 'replacement arthroplasty'/exp OR 'replacement arthroplasty' OR 'joint replacement'/exp OR 'joint replacement' OR 'joint replacements' OR 'replacement, joint'/exp OR 'replacement, joint' OR 'replacements, joint' OR 'replacement arthroplasties' OR 'total joint replacement'/exp OR 'total joint replacement' OR 'joint replacement, total' OR 'joint replacements, total' OR 'replacement, total joint' OR 'replacements, total joint' OR 'total joint replacements' OR 'total joint arthroplasty'/exp OR 'total joint arthroplasty' OR 'knee replacement'/exp OR 'knee replacement' OR 'arthroplasty, replacement, knee'/exp OR 'arthroplasty, replacement, knee' OR 'arthroplasties, replacement, knee' OR 'arthroplasty, knee replacement' OR 'knee replacement arthroplasties' OR 'knee replacement arthroplasty'/exp OR 'knee replacement arthroplasty' OR 'replacement arthroplasties, knee' OR 'knee arthroplasty, total'/exp OR 'knee arthroplasty, total' OR 'arthroplasty, total knee' OR 'total knee arthroplasty'/exp OR 'total knee arthroplasty' OR 'replacement, total knee' OR 'total knee replacement'/exp OR 'total knee replacement' OR 'knee replacement, total'/exp OR 'knee replacement, total' OR 'knee arthroplasty'/exp OR 'knee arthroplasty' OR 'arthroplasty, knee'/exp OR 'arthroplasty, knee' OR 'arthroplasties, knee replacement' OR 'replacement arthroplasty, knee' OR 'arthroplasty, replacement, partial knee' OR 'unicompartmental knee arthroplasty'/exp OR 'unicompartmental knee arthroplasty' OR 'arthroplasty, unicompartmental knee' OR 'knee arthroplasty, unicompartmental' OR 'unicondylar knee arthroplasty'/exp OR 'unicondylar knee arthroplasty' OR 'arthroplasty, unicondylar knee' OR 'knee arthroplasty, unicondylar' OR 'partial knee arthroplasty' OR 'arthroplasty, partial knee' OR 'knee arthroplasty, partial' OR 'unicondylar knee replacement' OR 'knee replacement, unicondylar' OR 'partial knee replacement' OR 'knee replacement, partial' OR 'unicompartmental knee replacement'/exp OR 'unicompartmental knee replacement' OR 'knee replacement, unicompartmental' OR 'hip replacement'/exp OR 'hip replacement' OR 'total hip replacement'/exp OR 'hip replacement, total' OR 'replacement, total hip' OR 'hip replacements, total' OR 'replacements, total hip' OR 'total hip replacements' OR 'total hip replacement' OR 'hip total replacement' OR 'hip total replacements' OR 'hip total joint replacement' OR 'hip total joint replacements' OR 'replacement, hip total joint' OR 'replacements, hip total joint' OR ' hip replacement, total joint' OR ' hip replacements, total joint' OR 'hip total replacement arthroplasty' OR 'hip total replacement arthroplasties' OR 'hip total arthroplasty' OR 'hip total arthroplasties' OR 'arthroplasty, replacement, hip' OR 'arthroplasties, replacement, hip' OR 'arthroplasty, hip replacement' OR 'hip replacement, arthroplasty' OR 'hip replacement, arthroplasties' OR 'hip prosthesis implantation' OR 'hip prosthesis implantations' OR 'implantation, hip prosthesis' OR 'implantations, hip prosthesis' OR 'prosthesis implantation, hip' OR 'prosthesis implantations, hip' OR 'hip replacement arthroplasty' OR 'replacement arthroplasties, hip' OR 'replacement arthroplasty, hip' OR 'arthroplasties, hip replacement' OR 'arthroplasty, hip replacement' OR 'hip replacement arthroplasties' OR 'total hip arthroplasty' OR 'total hip arthroplasties' OR 'total replacement hip arthroplasty' OR 'total replacement hip arthroplasties' | 112,009 |
|  | #13 Combine | #4 AND #13 | 402 |
|  | #14 Limit | #4 AND #13 AND [embase]/lim | 371 |

| **Database** | **Search** | **Search term** | **No. of studies (2019.5.10)** |
| --- | --- | --- | --- |
| Cochrane | #1 | [mh "Renal Insufficiency, Chronic"] | 5,920 |
|  | #2 | "Chronic Renal Insufficiencies":ti,ab,kw or "Renal Insufficiencies, Chronic":ti,ab,kw or "Chronic Renal Insufficiency":ti,ab,kw or "Kidney Insufficiency, Chronic":ti,ab,kw or "Chronic Kidney Insufficiency":ti,ab,kw or "Chronic Kidney Insufficiencies":ti,ab,kw or "Kidney Insufficiencies, Chronic":ti,ab,kw or "Chronic Kidney Diseases":ti,ab,kw or "Chronic Kidney Disease":ti,ab,kw or "Disease, Chronic Kidney":ti,ab,kw or "Diseases, Chronic Kidney":ti,ab,kw or "Kidney Disease, Chronic":ti,ab,kw or "Kidney Diseases, Chronic":ti,ab,kw or "Chronic Renal Diseases":ti,ab,kw or "Chronic Renal Disease":ti,ab,kw or "Disease, Chronic Renal":ti,ab,kw or "Diseases, Chronic Renal":ti,ab,kw or "Renal Disease, Chronic":ti,ab,kw or "Renal Diseases, Chronic":ti,ab,kw | 6,911 |
|  | #3 | [mh "Kidney Failure, Chronic"] | 4,343 |
|  | #4 | "Kidney Failure, Chronic":ti,ab,kw or "End-Stage Kidney Disease":ti,ab,kw or "Disease, End-Stage Kidney":ti,ab,kw or "End Stage Kidney Disease":ti,ab,kw or "Kidney Disease, End-Stage":ti,ab,kw or "Chronic Kidney Failure":ti,ab,kw or "End-Stage Renal Disease":ti,ab,kw or "Disease, End-Stage Renal":ti,ab,kw or "End Stage Renal Disease":ti,ab,kw or "Renal Disease, End-Stage":ti,ab,kw or "Renal Disease, End Stage":ti,ab,kw or "Renal Failure, End-Stage":ti,ab,kw or "End-Stage Renal Failure":ti,ab,kw or "Renal Failure, End Stage":ti,ab,kw or "Renal Failure, Chronic":ti,ab,kw or "Chronic Renal Failure":ti,ab,kw or "ESRD":ti,ab,kw | 10,302 |
|  | #5 Combine | {or #1-#4} | 14,809 |
|  | #6 | [mh "Arthroplasty, Replacement"] | 3,997 |
|  | #7 | "Arthroplasty, Replacement":ti,ab,kw or "Arthroplasties, Replacement":ti,ab,kw or "Joint Prosthesis Implantation":ti,ab,kw or "Implantation, Joint Prosthesis":ti,ab,kw or "Implantations, Joint Prosthesis":ti,ab,kw or "Joint Prosthesis Implantations":ti,ab,kw or "Prosthesis Implantation, Joint":ti,ab,kw or "Prosthesis Implantations, Joint":ti,ab,kw or "Replacement Arthroplasty":ti,ab,kw or "Joint Replacement":ti,ab,kw or "Joint Replacements":ti,ab,kw or "Replacement, Joint":ti,ab,kw or "Replacements, Joint":ti,ab,kw or "Replacement Arthroplasties":ti,ab,kw or "Total Joint Replacement":ti,ab,kw or "Joint Replacement, Total":ti,ab,kw or "Joint Replacements, Total":ti,ab,kw or "Replacement, Total Joint":ti,ab,kw or "Replacements, Total Joint":ti,ab,kw or "Total Joint Replacements":ti,ab,kw | 4,602 |
|  | #8 | "total joint arthroplasty":ti,ab,kw | 201 |
|  | #9 | [mh "Arthroplasty, Replacement, Knee"] | 2,264 |
|  | #10 | "Arthroplasty, Replacement, Knee":ti,ab,kw or "Arthroplasties, Replacement, Knee":ti,ab,kw or "Arthroplasty, Knee Replacement":ti,ab,kw or "Knee Replacement Arthroplasties":ti,ab,kw or "Knee Replacement Arthroplasty":ti,ab,kw or "Replacement Arthroplasties, Knee":ti,ab,kw or "Knee Arthroplasty, Total":ti,ab,kw or "Arthroplasty, Total Knee":ti,ab,kw or "Total Knee Arthroplasty":ti,ab,kw or "Replacement, Total Knee":ti,ab,kw or "Total Knee Replacement":ti,ab,kw or "Knee Replacement, Total":ti,ab,kw or "Knee Arthroplasty":ti,ab,kw or "Arthroplasty, Knee":ti,ab,kw or "Arthroplasties, Knee Replacement":ti,ab,kw or "Replacement Arthroplasty, Knee":ti,ab,kw or "Arthroplasty, Replacement, Partial Knee":ti,ab,kw or "Unicompartmental Knee Arthroplasty":ti,ab,kw or "Arthroplasty, Unicompartmental Knee":ti,ab,kw or "Knee Arthroplasty, Unicompartmental":ti,ab,kw or "Unicondylar Knee Arthroplasty":ti,ab,kw or "Arthroplasty, Unicondylar Knee":ti,ab,kw or "Knee Arthroplasty, Unicondylar":ti,ab,kw or "Partial Knee Arthroplasty":ti,ab,kw or "Arthroplasty, Partial Knee":ti,ab,kw or "Knee Arthroplasty, Partial":ti,ab,kw or "Unicondylar Knee Replacement":ti,ab,kw or "Knee Replacement, Unicondylar":ti,ab,kw or "Partial Knee Replacement":ti,ab,kw or "Knee Replacement, Partial":ti,ab,kw or "Unicompartmental Knee Replacement":ti,ab,kw or "Knee Replacement, Unicompartmental":ti,ab,kw | 7,510 |
|  | #11 | [mh "Arthroplasty, Replacement, Hip"] | 1,730 |
|  | #12 | "Arthroplasty, Replacement, Hip":ti,ab,kw or "Arthroplasties, Replacement, Hip":ti,ab,kw or "Arthroplasty, Hip Replacement":ti,ab,kw or "Hip Prosthesis Implantation":ti,ab,kw or "Hip Prosthesis Implantations":ti,ab,kw or "Implantation, Hip Prosthesis":ti,ab,kw or "Implantations, Hip Prosthesis":ti,ab,kw or "Prosthesis Implantation, Hip":ti,ab,kw or "Prosthesis Implantations, Hip":ti,ab,kw or "Hip Replacement Arthroplasty":ti,ab,kw or "Replacement Arthroplasties, Hip":ti,ab,kw or "Replacement Arthroplasty, Hip":ti,ab,kw or "Arthroplasties, Hip Replacement":ti,ab,kw or "Hip Replacement Arthroplasties":ti,ab,kw or "Hip Replacement, Total":ti,ab,kw or "Replacement, Total Hip":ti,ab,kw or "Hip Replacements, Total":ti,ab,kw or "Replacements, Total Hip":ti,ab,kw or "Total Hip Replacements":ti,ab,kw or "Total Hip Replacement":ti,ab,kw | 2,936 |
|  | #13 | "total hip arthroplasty":ti,ab,kw | 2,327 |
|  | #14 Combine | {or #6-#13} | 12,339 |
|  | #15 Combine | #5 and #14 | 22 |
